# Neoadjuvant chemotherapy combined with endocrine therapy for hormone receptor-positive breast cancer: A systematic review and meta-analysis

**DOI:** 10.1097/MD.0000000000035928

**Published:** 2023-11-17

**Authors:** Hong-Fang Ma, Jun Shen, Bin Xu, Jian-Guo Shen

**Affiliations:** a Department of Plastic Surgery, Sir Run Run Shaw Hospital, Zhejiang University School of Medicine, Hangzhou, China; b Department of Surgical Oncology, Sir Run Run Shaw Hospital, Zhejiang University School of Medicine, Hangzhou, China.

**Keywords:** breast cancer, hormone receptor-positive, meta-analysis, neoadjuvant chemotherapy, neoadjuvant endocrine therapy

## Abstract

**Background:**

This study aimed to conduct a comparative analysis of the efficacy and safety of neoadjuvant chemotherapy combined with endocrine therapy against the backdrop of single neoadjuvant chemotherapy or endocrine therapy, specifically in the context of hormone receptor-positive (HR+) breast cancer treatment.

**Methods:**

We conducted a thorough literature search across several databases, including China National Knowledge Infrastructure, Wanfang, Weipu, Chinese Journal Full-text Database, PubMed, Web of Science, Cochrane Library, and EMBASE, adhering to the guidelines outlined in the PRISMA statement. Our specific focus was on identifying randomized controlled trials that directly compared the combined approach of neoadjuvant chemotherapy and endocrine therapy with single chemotherapy or endocrine therapy in the context of treating HR+ breast cancer. Subsequently, we utilized statistical packages implemented in R software to perform comparative analyses of key clinical indicators, encompassing the complete response, objective response rate (ORR), disease control rate, pathological complete response (pCR), and adverse reactions.

**Results:**

A total of 11 randomized controlled trials, involving 1359 patients, all of whom met our inclusion criteria and were thus included in our comprehensive analysis. Within this cohort, 688 patients (50.63%) administered neoadjuvant chemotherapy combined with endocrine therapy (NCET), 642 patients (47.24%) received neoadjuvant chemotherapy (NCT) alone, while 29 patients (2.13%) underwent neoadjuvant endocrine therapy (NET) alone. The results of our meta-analysis revealed that NCET exhibited a statistically significant enhancement in both ORR and pCR (*P* < .05). Nonetheless, when compared to NCT or NET, NCET did not yield a significant impact on complete response, disease control rate, and safety (*P* > .05). In addition, NCET demonstrated a significant improvement in ORR among patients with HR+, HER2-negative breast cancer (*P* < .05). However, it was also linked to a heightened incidence of serious adverse reactions within this particular patient subgroup (*P* < .05).

**Conclusion:**

The combination of Neoadjuvant chemotherapy and endocrine therapy stands out as a significant contributor to enhancing the ORR and pCR for HR+ breast cancer patients. For breast cancer patients with HER2- status, NCET demonstrates a remarkable improvement in ORR but is also associated with the emergence of adverse reactions.

## 1. Introduction

The incidence of breast cancer, the most commonly diagnosed malignant tumor in women, has surpassed lung cancer in incidence and continues to increase by 0.3% per year.^[1,2]^ Surgical resection treatment is a common therapeutic approach for managing early-stage breast cancer in most patients. Currently, 2 main surgical options are available: mastectomy and breast-conserving surgery (BCS). A multicenter study conducted in an Asian population revealed that both BCS and mastectomy therapies resulted in similar survival rates.^[3]^ However, a systematic review and meta-analysis conducted by Zehra et al^[4]^ suggested that BCS offered superior health-related quality of life outcomes for breast cancer survivors compared to mastectomy. It’s worth noting that approximately 4 out of 5 breast cancer patients are diagnosed as hormone receptor-positive (HR+).^[5]^ For these patients, 2 key adjuvant-based treatment choices come into play, namely neoadjuvant chemotherapy (NCT) and endocrine therapy (NET). These treatments can be instrumental in facilitating BCS.^[6,7]^ For instance, research by Botty Van den Bruele et al^[8]^ showcased that NCT achieved nodal pathologic complete response in a majority of patients with occult primary breast cancer. Moreover, NET has been widely adopted for postmenopausal breast cancer, particularly for cases with estrogen receptor-positive (ER+), mirroring the role of NCT in HR+ breast cancer treatment.^[9]^

Previously conducted randomized controlled trials (RCTs) have yielded insights into the effectiveness of adjuvant chemotherapy involving cyclophosphamide, doxorubicin, and fluorouracil, combined with sequential endocrine therapy using tamoxifen, particularly in postmenopausal breast cancer patients with HR+ status.^[10]^ However, Toi et al^[11]^ have proposed an alternative treatment approach, demonstratin*g* the potential of adjuvant chemotherapy with fluoropyrimidines when paired with standard endocrine therapy for primary HR+ breast cancer. To date, numerous RCTs have been undertaken to assess the clinical efficacy, impact on quality of life, and the occurrence of associated adverse reactions in NCET for the treatment of HR+ breast cancer. However, these studies have produced inconclusive results, leaving the question of whether NCET offers superior efficacy in HR+ breast cancer treatment unanswered. In this current study, we have undertaken a comprehensive meta-analysis, aiming to compare clinical efficacy and the incidence of adverse reactions between NCET and NCT/NET. Our objective is to provide valuable insights and evidence to guide future clinical treatment decisions for HR+ breast cancer.

## 2. Methods

### 2.1. Search strategy

Our systematic literature search was meticulously conducted using a combination of subject terms and free words across both Chinese databases (China National Knowledge Infrastructure, Wanfang, Weipu, and Chinese Journal Full-text Database) and English databases (Pubmed, EMBASE, Web of Science, Cochrane Library). The search terms employed were as follows: “breast tumor,” “breast cancer,” “breast neoplasm,” “neoadjuvant endocrine therapy,” “neoadjuvant chemotherapy” and “neoadjuvant chemotherapy combined with endocrine therapy.” We scrutinized these databases comprehensively, encompassing studies available from their inception up to August 15th, 2023. To ensure the utmost inclusivity of relevant studies, we also diligently examined the reference lists of identified studies. Detailed information regarding our comprehensive search strategies for Pubmed can be found as follows:

**Table:** 

ID	Index and keyword terms
#1	(((((((((((“neoadjuvant endocrine therapy”[Title/Abstract]) OR (“neoadjuvant endocrine treatment”[Title/Abstract])) OR (“neoadjuvant chemotherapy”[Title/Abstract])) OR (“neoadjuvant therapy”[Title/Abstract])) OR (NET[Title/Abstract])) OR (NCT[Title/Abstract])) OR (“nonsteroidal aromatase inhibitor”[Title/Abstract])) OR (“anastrozole”[Title/Abstract])) OR (“tegafur”[Title/Abstract])) OR (“uracil”[Title/Abstract])) OR (“UFT”[Title/Abstract])) OR (“exemestane”[Title/Abstract])
#2	(((((((((((“neoadjuvant chemoendocrine therapy”[Title/Abstract]) OR (NCET[Title/Abstract])) OR (“anastrozole plus tegafururacil”[Title/Abstract])) OR (“Neoadjuvant endocrine therapy with exemestane”[Title/Abstract])) OR (“neoadjuvant chemotherapy with concurrent hormone therapy”[Title/Abstract])) OR (“neoadjuvant concurrent chemotherapy and letrozole”[Title/Abstract])) OR (“neoadjuvant chemotherapy combined with endocrine therapy”[Title/Abstract])) OR (“neoadjuvant chemotherapy plus endocrine therapy”[Title/Abstract])) OR (“neoadjuvant chemo-endocrine therapy”[Title/Abstract])) OR (“neoadjuvant chemo-endocrine treatment”[Title/Abstract])) OR (“neoadjuvant chemo-endocrine treatment”[Title/Abstract])) OR (“neoadjuvant chemo-endocrine treatment”[Title/Abstract])
#3	(((((((“breast tumor*”[Title/Abstract]) OR (“breast cancer”[Title/Abstract])) OR (“breast Neoplasm*”[Title/Abstract])) OR (“Mammary Cancer*”[Title/Abstract])) OR (“Cancer of Breast”[Title/Abstract])) OR (“Breast Carcinoma*”[Title/Abstract])) OR (“Cancer of the Breast”[Title/Abstract])) OR (“breast carcinoma”[Title/Abstract])
	#1 AND #2 AND #3

### 2.2. Inclusion and exclusion criteria

The study encompassed articles that adhered to the following criteria: patients included in the study were diagnosed with HR+ breast cancer; randomized controlled trials; NCET and NCT/NET were used in the experimental and control groups, respectively. Conversely, articles that met the following criteria were excluded from the study: literature reviews; duplicate articles; no full text version; had incomplete test data; and the participants without HR+ breast cancer.

### 2.3. Outcome measures

The outcome indicators included various key parameters, including clinical efficacy (complete response [CR], objective response rate [ORR], disease control rate [DCR], and pathological complete response [pCR]), and adverse reactions.

### 2.4. Data extraction and quality evaluation

Relevant information was meticulously extracted from eligible articles, encompassing critical details such as article title, authorship, publication date, total sample size, sample size within the experimental group and control group, specific methodologies employed, drug regimens utilized, clinical treatment timeline, pathological responses, and the occurrence of adverse reactions. Subsequently, we employed the Cochrane risk bias assessment tool to evaluate the overall quality of the included studies. This comprehensive evaluation took into account factors such as random sequence generation, allocation concealment, blinding of subjects and researchers, data completeness, determination of whether the studies were selective reports, and other biases. The results of this assessment were categorized into 3 groups: “Low risk,” “High risk,” and “Unclear risk.” It’s important to note that this assessment process was independently performed by 2 researchers.

### 2.5. Statistical analysis

The data underwent analysis using packages integrated into R version 4.3.1.^[12]^ We calculated *P*-valued and *I*^2^ using a heterogeneity test, utilizing these metrics to gauge the specificity of the literature included in our study. Studies with *P* ≥ .1 and *I*^2^ ≤ 50% were considered to have homogeneity and were therefore subjected to the fixed effects model. Otherwise, the random effects model was used. Our findings were presented in terms of risk ratios (RR) alongside their corresponding 95% confidence intervals (CI). To uphold the precision of our meta-analysis results, we conducted a sensitivity analysis with the R software, adopting a leave-one-out approach. Furthermore, we performed Egger’s regression and assessed funnel plots to detect and evaluate potential sources of bias. We considered *P* < .05 as the threshold for statistical significance.

## 3. Results

### 3.1. Basic characteristics of the included studies

**Figure 1.**
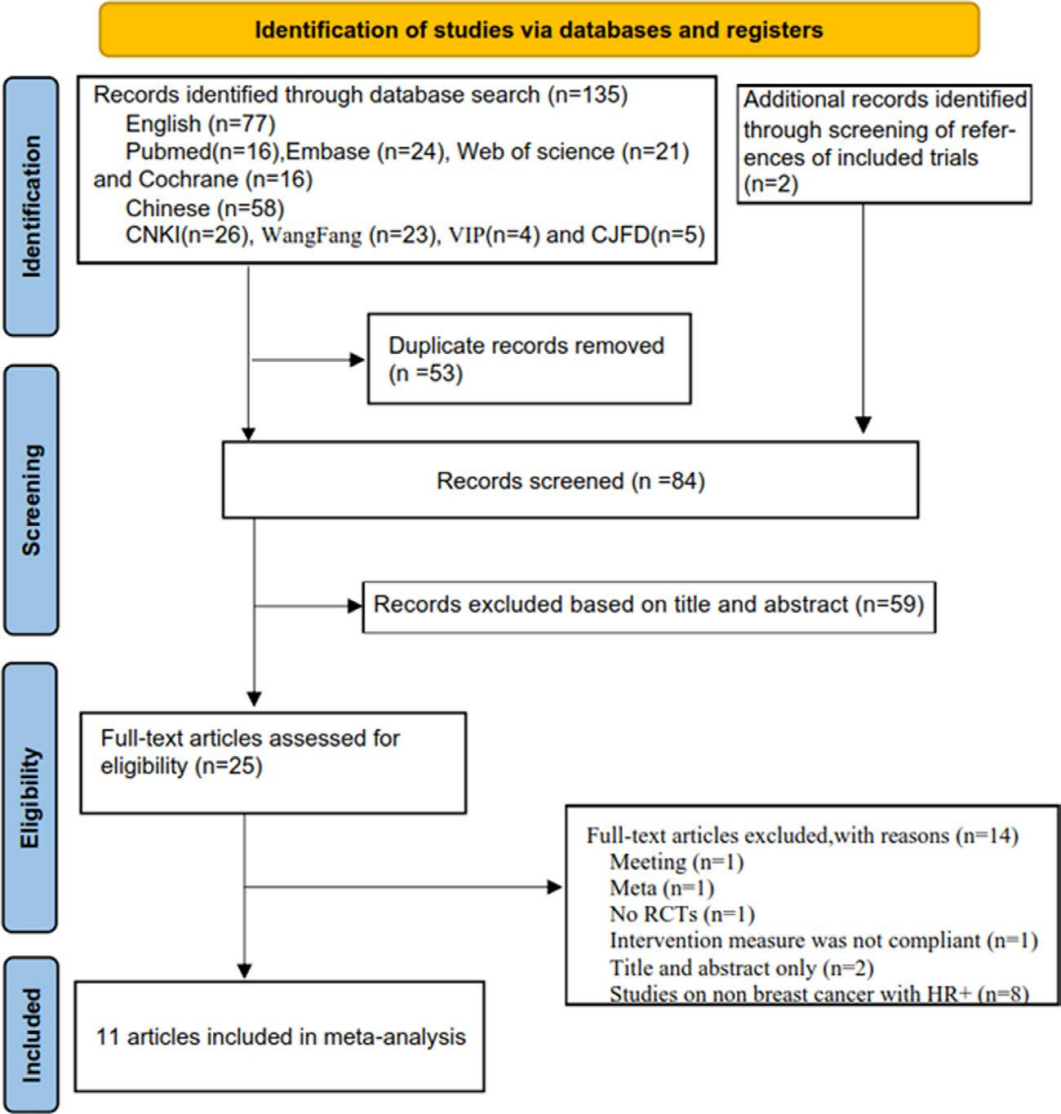
Document screening flow chart.

From the English databases, we initially identified a total of 77 articles, with 16 from PubMed, 24 from EMBASE, 21 from Web of Science, and 16 from Cochrane Library. Concurrently, in Chinese databases, we retrieved 26 articles from China National Knowledge Infrastructure, 23 from Wanfang, 4 from Weipu, and 5 from Chinese Journal Full-text Database. Following a meticulous screening process, we excluded 53 duplicate articles, while an additional 2 articles were sourced from the reference lists of the initially selected studies. Subsequently, our comprehensive search yielded a total of 84 articles. However, after a thorough evaluation of the title, abstract and full text of these titles, 73 articles were deemed ineligible for inclusion. Ultimately, we included 11 articles in our analysis, comprising 2 in Chinese and 9 in English. These selected studies collectively described a total of 1359 patients, distributed as follows: 688 in the NCET group, 642 in the NCT group, and 29 in the NET group. The process of literature search and study selection is presented in Figure 1, and the basic characteristics of the included studies are outlined in Table 1.

**Table 1:** Basic characteristics of included studies.

ID	Authors	Year	Research samples (T/C)	ER/PR	Her2	Time (d)	Interventions		Effect indexes
							NCET	NCT/NET	
1	Murray^[13]^	2022	81/41	ER+	Her2-	-	(Etrozole plus goserelin in pre-/perimenopausal women)+(Anthracycline and taxane)	(Anthracycline and taxane)	④
2	Matsunuma^[14]^	2020	36/34	ER+	Her2-	168	(Paclitaxel)+(doxorubicin, cyclophosphamide or epirubicin, cyclophosphamide)+(anastrozole or anastrozole plus leuprorelin)	(Paclitaxel)+(doxorubicin, cyclophosphamide or epirubicin, cyclophosphamide)	①②③④
3	Sato^[15]^	2019	38/15	ER+	-	168	(Exemestane)+(docetaxel–cyclophosphamide)	(Exemestane)	④⑤
4	Yu^[16]^	2019	125/124	ER+	Her2-	1825	(Epirubicin, Cyclophosphamide, paclitaxel)/(5-Fluorouracil, Epirubicin, Cyclophosphamide, Docetaxel)+(Letrozole, Leuprorelin)	(Epirubicin, Cyclophosphamide, paclitaxel)/(5-Fluorouracil, Epirubicin, Cyclophosphamide, Docetaxel)	①②③④
5	Nakayama^[17]^	2018	28/28	ER+ (>10%)	Her2-	168	(UFT)+(Anastrozole)	(UFT)	①②③⑤
6	Sato^[18]^	2018	42/14	ER+	Her2-	84	(Cyclophosphamide)+(Exemestane)	(Exemestae)	④⑤
7	Lu^[19]^	2017	56/36	ER+	-	168	(Docetaxel, Epirubicin,Cyclophosphamide)+(Goserelin Acetate/Letrozole)	(Docetaxel, Epirubicin, Cyclophosphamide)	②④
8	Li^[20]^	2017	100/100	ER+/PR+	-	56	(5-Fluorouracil, Epirubicin, Cyclophosphamide)+(Exemestane)	(5-Fluorouracil, Epirubicin, Cyclophosphamide)	①②③⑤
9	Kim^[13]^	2015	116/216	ER+/PR+	Her2-	-	(No clear)+(Goserelin)	(No clear)	①②③
10	Sugiu^[14]^	2015	16/12	ER+	Her2+/Her2-	180	(paclitaxel, 5-Fluorouracil, Epirubicin, Cyclophosphamide)+(Leuprorelin)	(paclitaxel, 5-Fluorouracil, Epirubicin, Cyclophosphamide)	①②③④
11	Mohammadianpanah^[15]^	2012	50/51	ER+, ER-d and other/PR+, PR- and other	Her2+/Her2-/other	63–91	(5-Fluorouracil, Doxorubicin, Cyclophosphamide)+(letrozole)	(5-Fluorouracil, Doxorubicin, Cyclophosphamide)	①②④

T: NCET; C: NCT/NET; ①: CR; ②: RR; ③: DCR; ④: pCR; ⑤: adverse reactions.

CR = complete response, DCR = disease control rate, NCET = neoadjuvant chemotherapy combined with endocrine therapy, NCT = neoadjuvant chemotherapy, NET = neoadjuvant endocrine therapy, pCR = pathological complete response, RR = risk ratios.

### 3.2. Risk bias assessment

**Figure 2.**
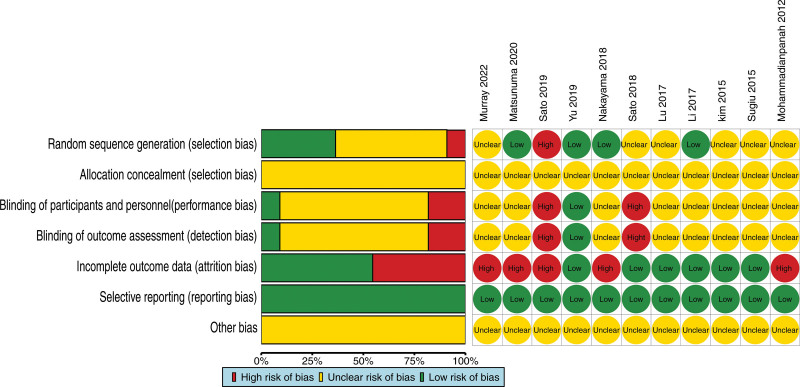
Diagram of risk assessment of bias in included studies.

We utilized the Cochrane Risk Assessment guidelines to evaluate the quality of the 11 articles included in the present study.^[13–23]^ Four articles^[14,18,21,22]^ provided clear descriptions of randomization methods, including computer-generated allowable blocks, minimization techniques and random number tables, warranting a “Low risk” rating. Six articles^[13,16,17,19,20,23]^ mentioned random grouping but did not specify the allocation method, resulting in an “Unclear risk” rating. One article^[15]^ employed patient Ki67 status for grouping, leading to a “High risk” rating. One article^[21]^ referred to blindness, and was considered “Low risk.” Two articles^[15,16]^ mentioned open label, and thus were rated “High risk.” In addition, articles that did not provide a detailed explanation were evaluated as “Unclear risk.” Five articles^[13–15,22,23]^ mentioned withdrawing, and were rated “High risk.” None of the articles mentioned allocation concealment or other biases, resulting in an “Unclear risk” rating. All the articles demonstrated no selective reporting, they were considered “Low risk” (Fig. 2).

### 3.3. Meta-analysis results

#### 3.3.1. Clinical efficacy.

##### 3.3.1.1. Complete response.

**Figure 3.**
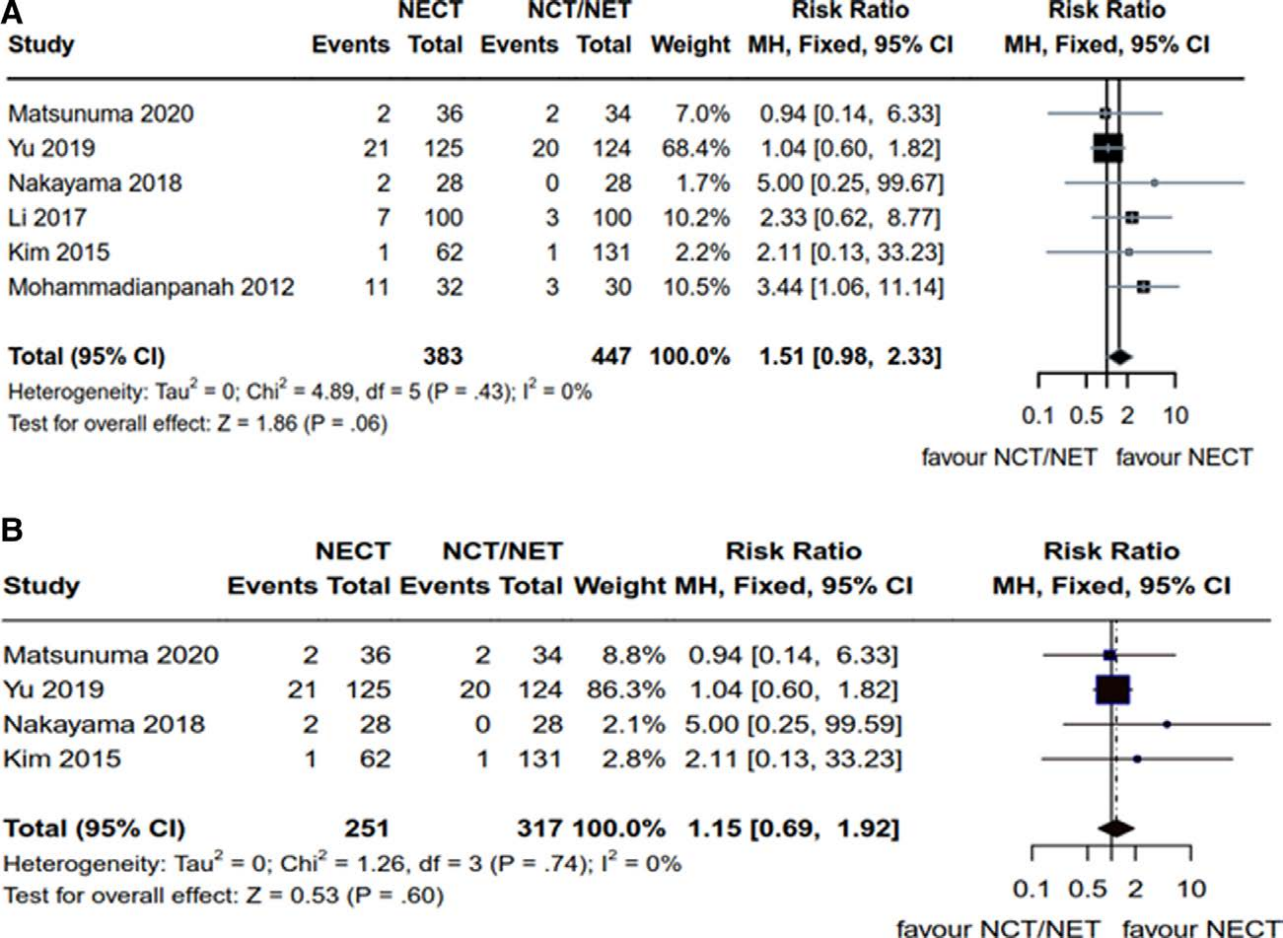
Comparative analysis of clinical CR of NCET and NCT/NET; (A) HR+ breast cancer patients (B) Her2- breast cancer patients. CR = complete response, HR+ = hormone receptor-positive, NCET = neoadjuvant chemotherapy combined with endocrine therapy, NCT = neoadjuvant chemotherapy, NET = neoadjuvant endocrine therapy.

Six articles^[14,18,19,21–23]^ reported on CR in HR+ breast cancer patients, encompassing a total of 830 patients, with 383 and 477 patients in the NCET and NCT/NET groups, respectively. Heterogeneity analysis yielded the following results: *P* = .43, *I*^2^ = 0% (*P* > .1, *I*^2^ < 50%), which justified the utilization of the fixed-effects model. Notably, the number of CR cases in breast cancer patients with HR+ status in NCET group was marginally higher than that in NET/NCT group; however, these differences did not achieve statistical significance [RR = 1.51, 95% CI (0.98, 2.33), *Z* = 1.86, *P* = .06] (Fig. 3A). Among these articles, 4 studies^[14,19,21,22]^ specified that the patients had Her2- breast cancer, involving a total of 568 patients (251 patients in the NCET group and 317 patients in the NCT/NET group). The heterogeneity results revealed the following: *P* = .74, *I*^2^ = 0% (*P* > .1, *I*^2^ < 50%), which warranted the application of the fixed-effects model. Importantly, there was no significant difference observed in the CR for Her2- breast cancer between the NCET group and the NET/NCT group [RR = 1.15, 95% CI (0.69, 1.92), *Z* = 0.53, *P* = 0. 60] (Fig. 3B).

##### 3.3.1.2. Objective response rate.

**Figure 4.**
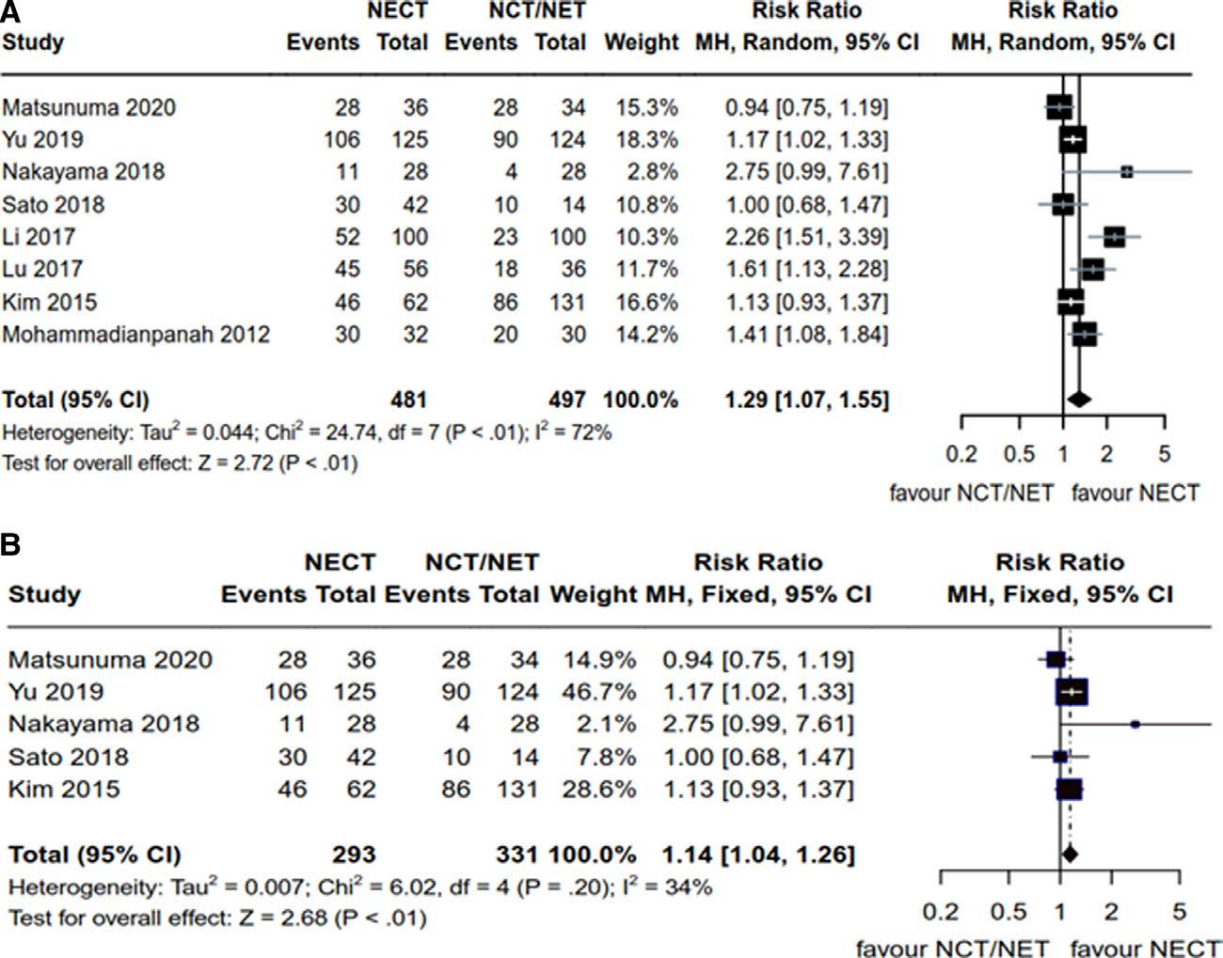
Comparative analysis of ORR of NCET and NCT/NET; (A) HR+ breast cancer patients (B) Her2- breast cancer patients. HR+ = hormone receptor- positive, NCET = neoadjuvant chemotherapy combined with endocrine therapy, NCT = neoadjuvant chemotherapy, NET = neoadjuvant endocrine therapy, ORR = objective response rate.

A total of 8 articles^[14,16–19,21–23]^ provided data on ORR in HR+ breast cancer patients, encompassing a collective cohort of 978 patients, with 481 and 497 in the NCET and NCT/NET groups, respectively. Heterogeneity analysis yielded the following results: *P* < .01, *I*^2^ = 72% (*P* < .1, *I*^2^ > 50%), which led to the adoption of the random-effects model. Notably, demonstrated superior efficacy compared to NCT/NET in terms of ORR for HR+ breast cancer patients [RR = 1.29, 95% CI (1.07, 1.55), *Z* = 2.72, *P* < .01] (Fig. 4A). Furthermore, 5 articles^[14,16,19,21,22]^ independently examined ORR efficacy in Her2- patients, encompassing a total of 624 Her2- breast cancer patients, with 293 and 331 in the NCET and NCT/NET groups, respectively. Heterogeneity results revealed the following: *P* = .20, *I*^2^ = 34% (*P* > .1, *I*^2^ < 50%), justifying the use of the fixed-effects model. In this context, NCET exhibited superior efficacy compared to NCT/NET in terms of ORR for Her2- breast cancer [RR = 1.14, 95% CI (1.04, 1.26), *Z* = 2.68, *P* < .01] (Fig. 4B).

##### 3.3.1.3. Disease control rate.

**Figure 5.**
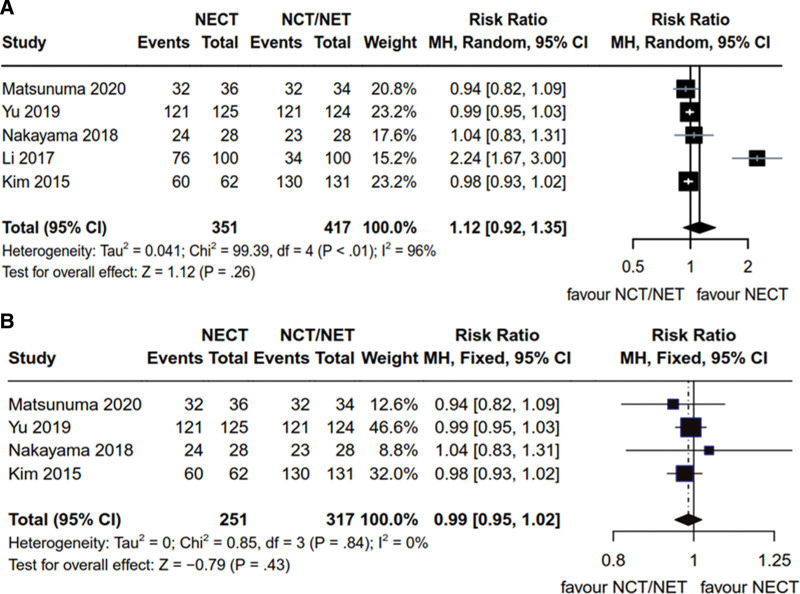
Comparative analysis of DCR of NCET and NCT/NET; (A) HR+ breast cancer patients (B) Her2- breast cancer patients. DCR = disease control rate, HR+ = hormone receptor-positive, NCET = neoadjuvant chemotherapy combined with endocrine therapy, NCT = neoadjuvant chemotherapy, NET = neoadjuvant endocrine therapy.

In this meta-analysis, a total of 5 articles^[14,18,19,21,22]^ reported on DCR in breast cancer patients with HR+ status. These studies collectively enrolled 768 patients, with 351 and 417 were in the NCET and NCT/NET groups, respectively. Heterogeneity analysis yielded the following results: *P* < .01, *I*^2^ = 96% (*P* < .1, *I*^2^ > 50%), necessitating the application of the random-effects model. It was observed that the DCR for HR+ breast cancer in the NCET group was slightly higher than that in the NCT/NET group, but the difference did not reach statistical significance [RR = 1.12, 95% CI (0.92, 1.35), *Z* = 1.12, *P* = .26] (Fig. 5A). Among these articles, 4 studies^[14,19,21,22]^ specifically addressed Her2- patients, comprising a total of 568 enrolled individuals (251 in the NCET group and 317 in the NCT/NET group). Heterogeneity results were as follows: *P* = .84, *I*^2^ = 0% (*P* > .1, *I*^2^ < 50%), thus warranting the use of the fixed-effects model. In this context, there was no statistically significant difference observed between NCET and NCT/NET in terms of the treatment efficacy for DCR in Her2- breast cancer [RR = 0.99, 95% CI (0.95, 1.02), *Z* = −0.79, *P* = 0. 43] (Fig. 5B).

##### 3.3.1.4. Pathological complete response.

**Figure 6.**
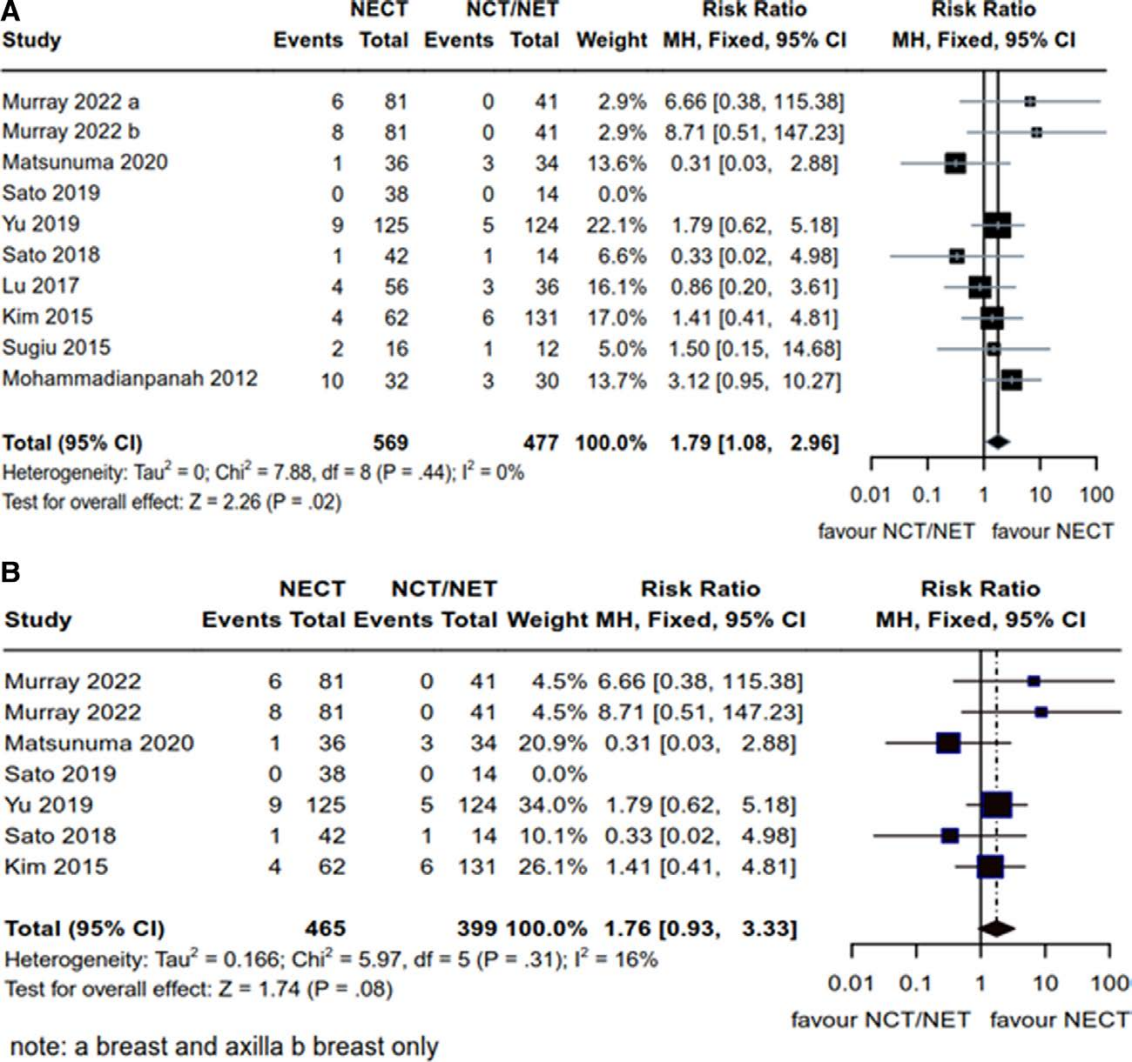
Comparative analysis of pathological complete remission rate of NCET and NCT/NET; (A) HR+ breast cancer patients (B) Her2- breast cancer patients. HR+ = hormone receptor-positive, NCET = neoadjuvant chemotherapy combined with endocrine therapy, NCT = neoadjuvant chemotherapy, NET = neoadjuvant endocrine therapy.

Nine articles in total^[13–17,19–21,23]^ provided data on pCR in breast cancer patients with HR+ status. These studies enrolled a total of 1046 patients, with 569 in the NCET group and 477 in the NCT/NET group. Heterogeneity results were as follows: *P* = .44, *I*^2^ = 0% (*P* > .1, *I*^2^ < 50%), justifying the use of fixed-effects model. Notably, significant differences were observed between NCET and NCT/NET regarding the treatment efficacy for pCR in HR+ breast cancer patients [RR = 1.79, 95% CI (1.08, 2.96), *Z* = 2.26, *P* = .02] (Fig. 6A). Among these articles, 6 studies,^[13–16,19,21]^ specifically addressed Her2- patients, comprising a total of 864 enrolled individuals (465 in the NCET group and 399 in the NCT/NET group). Heterogeneity results were as follows: *P* = .31, *I*^2^ = 16% (*P* > .1, *I*^2^ < 50%), which led to the application of the fixed-effects model. In this context, NCET demonstrated a higher pCR rate compared to the NCT/NET group; however, the difference was not statistically significant in the treatment of Her2- breast cancer [RR = 1.76, 95% CI (0.93, 3.33), *Z* = 1.74, *P* = .08] (Fig. 6B).

#### 3.3.2. Adverse reactions.

**Figure 7.**
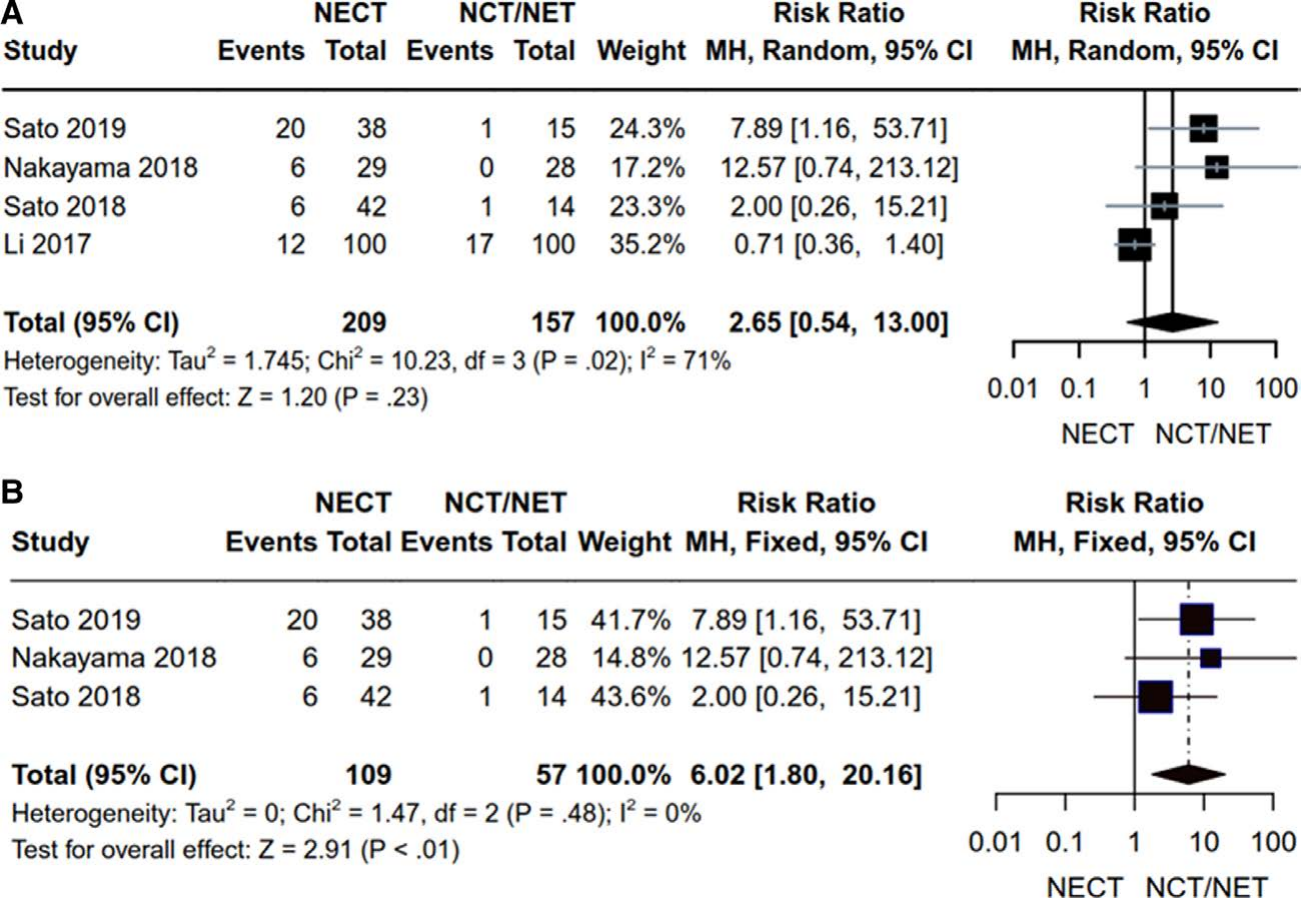
Comparative analysis of side-effects of NCET and NCT/NET; (A) HR+ breast cancer patients (B) Her2- breast cancer patients. HR+ = hormone receptor-positive, NCET = neoadjuvant chemotherapy combined with endocrine therapy, NCT = neoadjuvant chemotherapy, NET = neoadjuvant endocrine therapy.

Four articles^[15,16,18,22]^ reported on the incidences of adverse reactions (Grade ≥ 3) in HR+ breast cancer patients. These studies collectively enrolled a total of 366 patients, with 209 the NCET group and 157 in the NCT/NET group. Heterogeneity analysis yielded the following results: *P* = .02, *I*^2^ = 71% (*P* < .1, *I*^2^ > 50%), which led to the utilization of the random-effects model. Remarkably, no significant difference was observed between NCET and NCT/NET regarding the treatment of adverse reactions in HR+ breast cancer patients [RR = 2.65, 95% CI (0.54, 13.00), *Z* = 1.20, *P* = .23] (Fig. 7A). Furthermore, 3 articles^[15,16,22]^ independently analyzed adverse reactions (Grade ≥ 3) in Her2- patients. These studies encompassed a total of 166 Her2- breast cancer patients, with 109 in the NCET group and 57 in the NCT/NET group. Heterogeneity results were as follows: *P* = .00, *I*^2^ = 48% (*P* > .1, *I*^2^ < 50%), leading to the application of the fixed-effects model. In this context, adverse reactions caused by NCET were significantly higher than those caused by NCT/NET in the treatment of Her2- breast cancer [RR = 6.02, 95% CI (1.80, 20.16), *Z* = 2.91, *P* < .01] (Fig. 7B).

### 3.4. Publication bias

**Figure 8.**
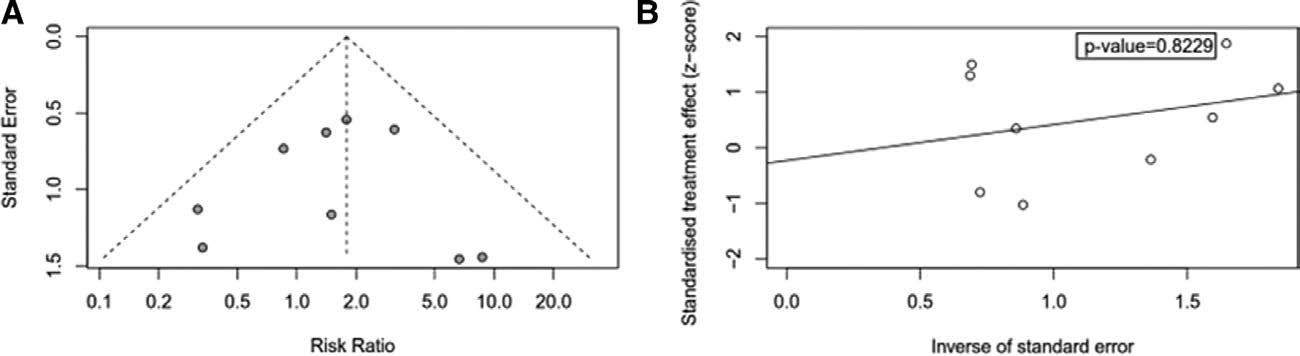
Funnel diagram.

Nine articles in total^[13–17,19–21,23]^ reported on pCR in HR+ breast cancer patients. To assess publication bias, we generated an inverted funnel plot using R packages and conducted Egger’s test analysis. The resulting inverted funnel chart displayed a generally symmetrical shape, and the results of Egger’s test were not statistically significant (*P* = .8229), indicating the presence of minimal publication bias (Fig. 8).

### 3.5. Sensitivity analysis

Sensitivity analysis was conducted for 3 key indicators: ORR, DCR, and adverse reactions (*I*^2^ > 50% and *P* < .1), utilizing a leave-one-out approach as detailed in Table 2. The outcomes demonstrated that, even after eliminating the item with the highest heterogeneity, the conclusions regarding ORR and DCR remained in line with the overall meta-analysis results. However, it is noteworthy that the results for adverse reactions diverged from the meta-analysis findings after the exclusion of the most influential outlier. In summary, the meta-analysis results for ORR and DCR are deemed stable and reliable.

**Table 2:** Sensitivity analysis.

Index	*I* ^2^	*P*	Meta			After deletion	Sensitivity analysis		
			RR	95% CI	*P*		RR	95% CI	*P*
ORR	72%	<.01	1.29	1.07, 1.55	<.01	Omitting Li 2017	1.19	1.03, 1.37	.02
DCR	96%	<.01	1.12	0.92, 1.35	.26	Omitting Li 2017	0.98	0.95, 1.01	.29
Adverse reactions	71%	.02	2.65	0.54, 13.00	.23	Omitting Li 2017	5.13	1.47, 17.92	.01

CI = confidence intervals, DCR = disease control rate, ORR = objective response rate, RR = risk ratios.

## 4. Discussion

The utilization of neoadjuvant treatment in breast cancer management has significantly improved breast-conserving rates and operability, establishing it as the gold standard for locally advanced breast cancer patients.^[24]^ Tailoring appropriate NCT regimens to different molecular subtypes of breast cancer has led to notable improvements in clinical outcomes and patient cases.^[25]^ However, approximately 70% of breast cancer cases are diagnosed as HR+ and HER2- subtypes, which are less responsive to NCT. Given the limited benefits of NCT in ER+ breast cancer, researchers have turned their attention to exploring alternative adjuvant endocrine therapies. HR+ breast cancer, being more reliant on estrogen, demonstrates greater responsiveness to endocrine therapy through estrogen deprivation. In recent years, clinical studies have emphasized the potential of endocrine drugs, particularly third-generation aromatase inhibitors, as a viable preoperative treatment option for stage II to III HR+ breast cancer in women.^[26]^ A consensus has emerged in favor of NET over NCT due to its improved tolerability and lower toxicity. However, questions remained regarding the clinical efficacy of integrating NCT into NET for HR+ breast cancer treatment. Our findings confirm that HR+ breast cancer patients undergoing NCET were more likely to achieve higher ORR and pCR compared to those receiving NCT or NET alone. Nevertheless, there were no significant differences observed in CR, DCR, or adverse reactions.

The results of our present study have demonstrated that NCET could significantly enhance the ORR in HR+ breast cancer patients when compared to NCT/NET. This observation aligns with existing evidence indicating that the NCET is effective in improving the clinical response rate in advanced HR+ breast cancer cases.^[27,28]^ This heightened efficacy might be attributed to the synergistic anti-tumor activity resulting from the enhancement of endocrine therapy when combined with metronomic chemotherapy. Importantly, prior research has also underscored the benefits of NCET, particularly when employing UFT (uracil and tegafur) in conjunction with tamoxifen as a 2-year postoperative adjuvant therapy for breast cancer patients with ER+ status.^[23]^ NCET has been shown to exert a broad-spectrum antitumor effect and offers favorable efficacy when compared to monotherapy with endocrine therapy alone. However, it’s noteworthy that although our study found that NCET leads to higher clinical parameters in terms of CR and DCR compared to NET or NCT alone, these differences did not reach statistical significance. As such, NCET primarily appears to enhance the clinical efficacy of HR+ breast cancer patients, particularly in terms of ORR.

In preoperative settings, the pCR rate can be used as a replace marker to predict event-free or overall survival in luminal HER2- breast cancer patients.^[29]^ The relationship between pCR and the survival of BC subtypes has been the topic of extensive investigation in the medical literature. A positive correlation between pCR and survival in triple negative and HER2+ breast cancer has been reported, but the correlation is unclear in breast cancer with luminal type. Von Minckwitz et al^[29]^ produced that there is no correlation between pCR and event free survival rate. Cortazar et al^[30]^ found that the pCR rate was 7.5% in HER2- luminal subtype grade 1 to 2 tumors by conducting a meta-analysis of 12 randomized controlled trials, whose result is similar to the above. These findings indicate that Luminal A type breast cancer has a lower pCR rate compared with other subtypes, and pCR cannot replace EFS. Therefore, the main purpose of neoadjuvant therapy, especially luminal type A, seems to be to shrink the tumor and allow for small-scale surgery, rather than improving pCR and providing survival advantages. According to our meta analysis results, the number of pCR in breast cancer with HR+ in NCET group was much higher than that in NET or NCT group, and there was significant difference.

Our study also found that NCET does not increase adverse events in breast cancer patients with HR+, which indicates that concurrent hormone therapy and chemotherapy appear an attractive treatment strategy. To date, only a few clinical studies have explored the safety of endocrine therapy and neoadjuvant chemotherapy in the treatment of HR+ breast cancer, whose conclusions that NCET toxicity is within an acceptable range, are consistent with the conclusion of our study.^[14,31]^ Due to the limited number of RCTs, inaccurate conclusions were obtained. Therefore, more RCTs are needed in the later stage to compare the security of NCET with NET or NCT.

This study had some limitations. Firstly, except for 3 articles^[18,21,22]^ that specified the specific random allocation method used, 5^[16,17,19,20,23]^ mentioned “random,” and lacked a description of the specific allocation method while 1 article is assigned based on the patient’s Ki67 status. This may have led to false positive results. Secondly, our sample size (which comprised only 11 RCTs) was small, thus may have caused some deviations. Thirdly, only articles in the Chinese and English languages were included, thus there may have been a certain degree of language bias. Fourthly, conference abstracts were not included. Lastly, there was only a single comparison of the differences between NCET and NCT/NET for the treatment of breast cancer with HR+.

## 5. Conclusion

In conclusion, our study clearly demonstrated that the NCET exhibited superior clinical efficacy, as evidenced by higher ORR and pCR, all while maintaining a comparable safety profile without an increase in adverse reactions. This therapeutic approach proves effective in optimizing neoadjuvant treatment and enhancing the potential for breast conservation. Overall, our findings provide valuable insights that can guide the development of future strategies for the clinical treatment of breast cancer.

## Author contributions

**Conceptualization:** Jian-Guo Shen.

**Data curation:** Bin Xu.

**Writing – original draft:** Hong-Fang Ma.

**Writing – review & editing:** Jun Shen.
